# Shear bond strength of metal brackets to ceramic surfaces using a universal bonding resin

**DOI:** 10.4317/jced.54175

**Published:** 2018-08-01

**Authors:** Roya Naseh, Maryam Afshari, Fereshteh Shafiei, Nima Rahnamoon

**Affiliations:** 1 Associate Professor, Dental Caries Prevention Research Center, Qazvin University of Medical Sciences, Qazvin, Iran; 2 Post Graduated Student,Dental Caries Prevention Research Center, Qazvin University of Medical Sciences, Qazvin, Iran. Assistant Professor, Department of Orthodontics, Lorestan University of Medical Sciences, Khorramabad, Iran; 3 Professor,Oral and Dental Disease Research Center, Department of Operative Dentistry,Shiraz University of Medical Sciences, Shiraz, Iran; 4 Assistant Professor, Department of Orthodontics, Lorestan University of Medical Sciences, Khorramabad, Iran

## Abstract

**Background:**

Assure Plus is a recently introduced universal adhesive with the ability to bond to various restorations. This study compared the shear bond strength of brackets bonded to two types of ceramics using conventional bonding agent and Assure Plus. Surface damage caused by debonding was also evaluated.

**Material and Methods:**

In this *in vitro* study, 40 feldspathic and lithium disilicate ceramic discs were sandblasted, etched with 9.6% hydrofluoric acid and divided into two groups. In group 1, silane was applied and air-dried followed by application of Transbond XT primer, which was light-cured. In group 2, Assure Plus was applied and air-dried. In both groups, maxillary central incisor brackets were bonded. After incubation in distilled water at 37°C for 24 hours and 2000 thermal cycles, bond strength was measured using a universal testing machine, and the adhesive remnant index (ARI) and failure modes were determined. ANOVA and LSD tests were used to compare bond strength values; chi-squared test was used to compare ARI scores.

**Results:**

Bracket bond to lithium disilicate by Assure Plus was significantly stronger than that to Feldspathic porcelain (*P*=0.041). Only in the Assure Plus/lithium disilicate group did some adhesive remain on the surface following debonding (40% of samples, *P*<0.05). Cohesive porcelain fracture had the lowest frequency in the lithium disilicate/Assure Plus group.

**Conclusions:**

Assure Plus provided high bond strength between ceramic and brackets and minimized damage to lithium disilicate ceramic during debonding. Assure Plus is recommended for use in orthodontic treatment of adults with ceramic restorations.

** Key words:**Adhesives, ceramics, dental bonding, shear bond strength.

## Introduction

Successful orthodontic treatment, in the presence of ceramic restorations, requires sufficiently high and durable bond between brackets and ceramic surfaces. There are two main challenges in this respect. The first challenge is to achieve 6‒10 MPa of bond strength to decrease bracket bond failure during the course of treatment. Bracket bond failure prolongs the course of treatment and wastes time (1). The second challenge is to maintain optimal esthetics and function of ceramic restorations following bracket debonding. In other words, bond strength should not be too high to damage the porcelain surface during debonding (2).

Since glazed porcelain is not suitable for bracket bonding, the literature suggests a wide range of surface treatments for different types of porcelain (3). Sandblasting, as a mechanical conditioning for the porcelain surface, removes the glaze and increases surface roughness, creating micromechanical retention (3,4). Hydrofluoric acid (HF) etching, as a chemical conditioning, is capable of partially dissolving the glassy phase, increasing surface microroughness (3-5). Following increased contact surface area through the two conditioning methods, silane improves the wettability of the surface and creates a covalent chemical bond between the silica of the porcelain and the organic groups of the bonding resin (5,6). Combination of silane with sandblasting or HF etching was reported to increase bracket bonding to porcelain significantly (3-5,7,8). Sandblasting followed by HF, followed by silanization and application of bonding resin, has been recommended as an optimal protocol (9).

Various types of ceramic restorations could be involved in bracket bonding. Silica-based ceramics, including feldspathic in ceramo-metal crowns, leucite-reinforced and lithium disilicate glass ceramics, are commonly used in adults needing orthodontic treatment. The different microstructure and processing techniques might influence bracket bond strength to different ceramics (10). Alhaija and Al-Wahadni compared the shear bond strength of stainless steel brackets to different ceramic surfaces and assessed the failure modes. The shear bond strength values of feldspathic porcelain and In-Ceram were almost equal while IPS-Impress showed the lowest shear bond strength value, which was significantly lower than that of feldspathic porcelain and In-Ceram surfaces. The failure mode in the feldspathic group was adhesive at the porcelain‒adhesive interface while it was adhesive at the bracket‒adhesive interface in the two other ceramic groups (11). Sarac *et al.* demonstrated a higher bracket bonding to leucite-reinforced ceramic than that of feldspathic and lithium disilicate ceramics. Adhesive failure between ceramic and composite was observed for all the groups in their study (10).

Assure Plus All Surface bonding resin is a recently introduced universal adhesive (launched in 2015). The manufacturer claims that it provides adequately high bond strength to normal as well as hypo-calcified and fluorosed enamel, primary teeth and dentin. It can also bond to irregular metal surfaces such as amalgam, gold or stainless steel and porcelain, zirconia, composite restorations, temporary restorations or acrylic pontics. The ability to bond to porcelain and zirconia differentiates this bonding agent from its previous generation, i.e. Assure Universal bonding agent. Moreover, Assure Plus can be polymerized by light-curing, chemical curing and dual-curing systems (12).

The instructions provided by the manufacturer for bonding to porcelain include sandblasting the surface, acid etching, rinsing and drying, followed by the application of a layer of Assure Plus and gentle air-drying with air spray. The bracket is then bonded by use of composite resin (12). Using this adhesive, silanization is no longer required compared to the use of conventional and Assure bonding agents. This study aimed to assess and compare the shear bond strength of orthodontic brackets to feldspathic porcelain and lithium disilicate ceramic using a conventional bonding agent and Assure Plus universal adhesive. The damage to the porcelain surface during bracket debonding was also assessed.

## Material and Methods

This *in vitro* study was conducted on 20 discs of feldspathic porcelain (Vita, Zahnfabrilic, Sackingen, Germany) and 20 discs of lithium disilicate ceramic (IPS E-max, Ivoclar, Vivadent, Liechtenstein), measuring 2 mm in thickness and 7 mm in diameter. Sample size was calculated at n=8 in each subgroup, considering α=0.05, β=0.2, S1=0.8, S2=0.5 and study power=0.8, using sample size calculation formula. For further accuracy, 10 samples were fabricated for each subgroup. The samples were fabricated by a skilled technician (A.M.) according to the manufacturers’ instructions and glazed. Feldspathic porcelain discs were made through condensation technique and backing under vacuum at 920°C and IPS E-max heat-pressed under vacuum at 910°C. After fabrication, the samples were visually inspected under a stereomicroscope (×10 magnification) to ensure absence of cracks and defects. The samples in the two groups were randomly divided into two subgroups of test and control. For ceramic surface preparation, the samples were sandblasted with micro-etcher (Pie Me SRL, Longigo-Veneza, Italy) using 50-μ alumina particles with 80-psi pressure for five seconds from a 10-mm distance and were then etched with 9.6% hydrofluoric acid (Pulpdent, USA) for two minutes, rinsed with water and dried with oil-free air spray.

In the control subgroups, first one layer of silane (Rely X Ceramic Primer, 3M ESPE, St. Paul, MN, USA) was applied on the ceramic surface and air-dried with gentle air spray. One layer of Transbond Primer (XT adhesive, Primer, 3M, ESPE, MN, USA) was applied on the samples and light-cured for 10 seconds using an LED light-curing unit (Mectron Dental, Carasco, Italy) calibrated by a radiometer with a light intensity of 1000 mW/cm2.

Maxillary central incisor edgewise brackets with 0.018-inch slot (GAC International, Bohemia, NY) and 11.26-mm2 base area were used. Transbond XT composite (3M ESPE, St. Paul, MN, USA) was applied to the base of the brackets which were placed at the center of the prepared ceramic surface. The bracket was compressed on the ceramic surface, and excess composite around the bracket was removed by the tip of an explorer. Light-curing was carried out for 40 seconds (10 seconds from each of the mesial, distal, buccal and lingual aspects) using a light-curing unit.

**Figure 1:**
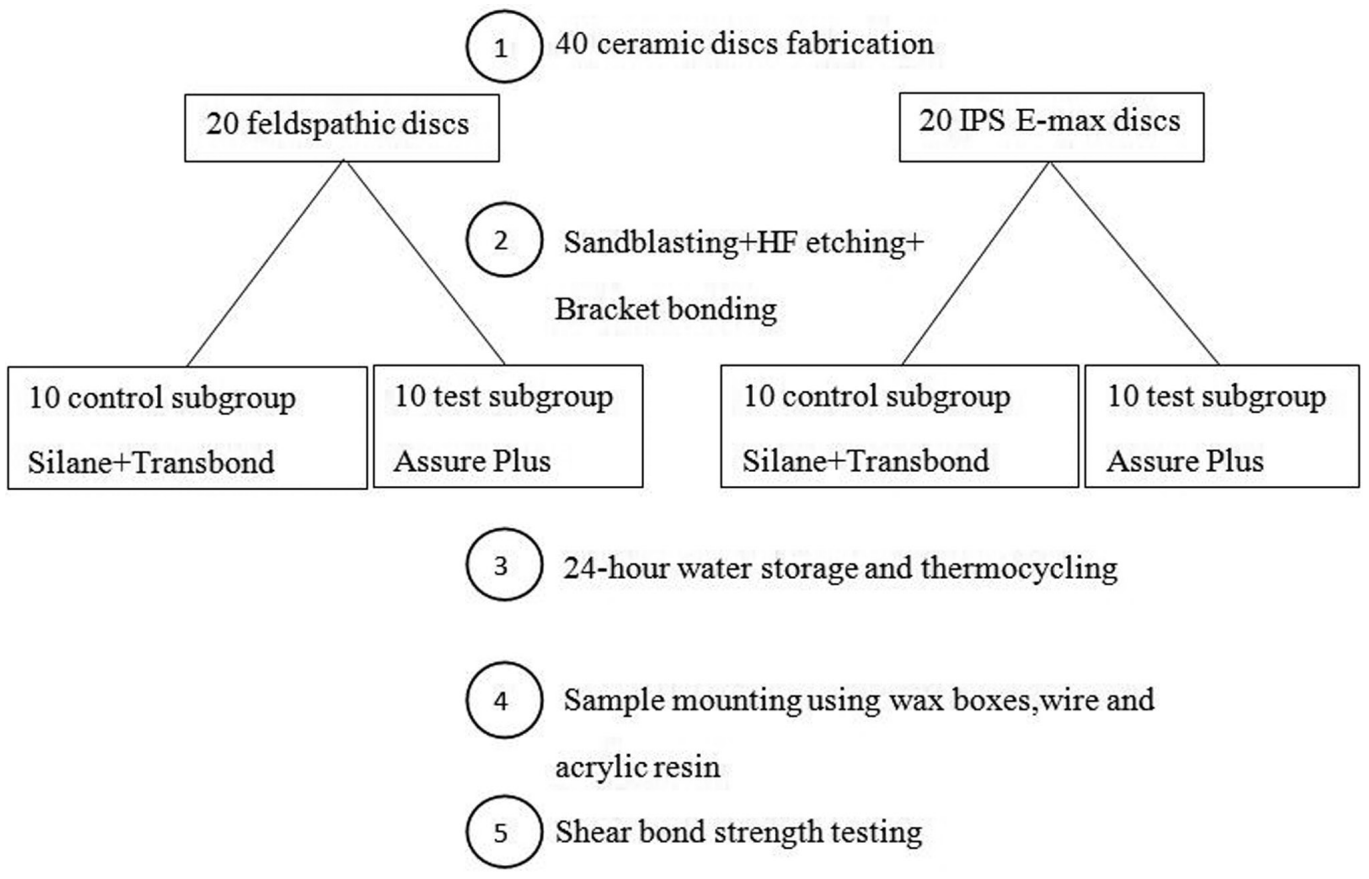
Schematic view of the processing steps of the experiment.

In the test subgroups, Assure Plus universal adhesive (Reliance, Itasca, IL, USA) was applied on the prepared ceramic surface and gently air-dried with air spray. The next steps were performed as in the previous group without the use of composite primer. All the experimental steps in the four groups are summarized in Figure 1. The bonded samples were incubated (Dorsa, Tehran, Iran) in distilled water at 37°C for 24 hours and were then subjected to 2000 thermal cycles at 5°/55°C (20 seconds in cold bath, 20 seconds in warm bath and 20 seconds of transfer time).

**Figure 2:**
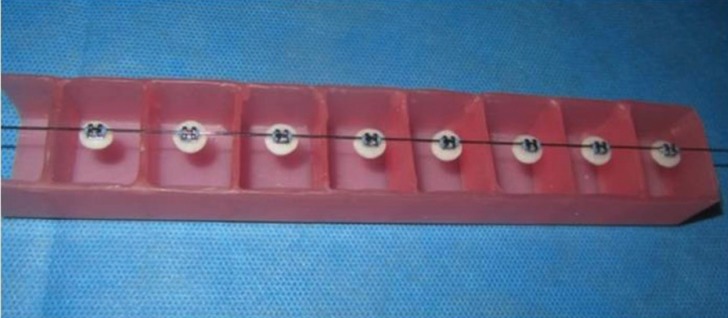
Wax boxes with wire ratchet for mounting the disks.

**Figure 3:**
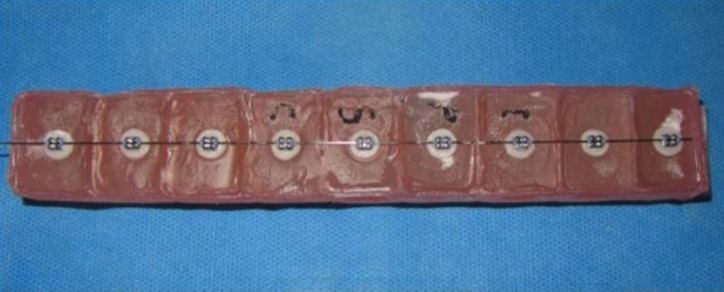
Mounted disks for bracket bond strength testing.

For mounting of the samples in acrylic resin for bond strength testing, wax boxes were fabricated and the brackets were placed on top of the boxes (10 per each row) using 0.017×0.025-inch wire ratchets using elastomeric ligatures in such a way that they were parallel to the longitudinal margins of the box (Fig. 2). Auto-polymerizing acrylic resin was poured into the box up to the upper margin of ceramic discs. The samples were embedded in the acrylic resin (Fig. 3). The contact of acrylic resin and bracket was prevented as such and a proper stub was obtained for placement of samples in the universal testing machine. To measure the shear bond strength, the samples were placed in the universal testing machine (Z020; ZwickRoell GmbH & Co., Ulm, Germany) and subjected to shear loads at a crosshead speed of 1 mm/minute. The load at fracture was recorded in Newtons. By dividing the load in N by the cross-sectional area of bracket (mm2), shear bond strength of brackets was calculated in MPa. The technician (S.H.) who carried out the procedure was blinded to the group allocation of samples. The samples were evaluated under a stereomicroscope (Carl Zeiss, Germany) at ×10 magnification to determine the mode of failure. The ARI score was also calculated based on the amount of adhesive remaining on the surface using a four-point scale as follows:

Score 0: No adhesive remaining on the surface

Score 1: Less than 50% of adhesive remaining on the surface

Score 2: More than 50% of adhesive remaining on the surface 

Score 3: The entire adhesive remaining on the surface

Failure modes were determined as follows:

Cohesive in the porcelain (CP): Fracture or crack within the porcelain 

Adhesive at the porcelain interface (AP): No fracture occurred in the porcelain. No adhesive remained on the porcelain surface; the entire adhesive remained on the bracket base.

Cohesive in resin (CR): Some resin remained on the porcelain surface and some on the bracket base.

Adhesive at the bracket interface (AB): The entire resin remained on the porcelain surface.

The shear bond strength values of orthodontic brackets to ceramic surfaces were statistically analyzed using ANOVA. LSD post hoc tests were used for pair-wise comparisons of the groups in terms of bond strength. Chi-squared test was applied to compare the groups in terms of ARI scores.

## Results

The means shear bond strength values and standard deviations in the four groups are presented in  Table 1. The highest bond strength was found in IPS ceramic samples following the use of Assure Plus, while the lowest bond strength was noted in feldspathic porcelain with the use of conventional bonding agent. According to Kolmogorov-Smirnov test, the data had normal distribution. According to ANOVA, significant differences were noted in shear bond strength of the groups (*P*=0.041,  Table 1).

**Table 1:**
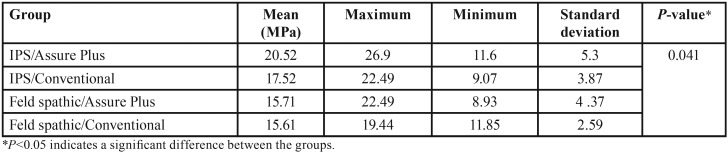
The mean shear bond strength in the groups.

LSD post hoc tests showed significant differences in terms of shear bond strength of brackets between IPS/Assure Plus and feldspathic/Assure Plus (*P*=0.014) and feldspathic/conventional adhesive (*P*=0.012) but the difference between the two groups of IPS/Assure Plus and IPS/conventional adhesive was not significant (*P*=0.11). No significant differences were noted between IPS/conventional adhesive and feldspathic/Assure Plus (*P*=0.33), IPS/conventional adhesive and feldspathic/conventional adhesive (*P*=0.31) or between feldspathic/Assure Plus and feldspathic porcelain/conventional adhesive (*P*=0.95).

 Table 2 shows the frequency of ARI scores in the groups. Based on the results of chi-squared test, there were significant differences in ARI scores among the groups (*P*<0.05). The ARI score of IPS/Assure Plus was significantly different from that in the other groups, and the amount of adhesive remaining on the ceramic surface was higher in IPS/Assure Plus compared to that in other groups.

**Table 2:**
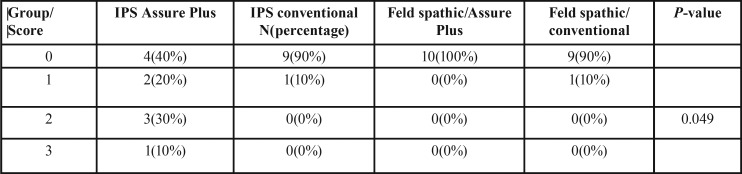
The frequency of adhesive remnant index scores in the groups.

None of the samples showed adhesive failure at the porcelain‒resin interface. The highest frequency of cohesive failure in the porcelain was noted in feldspathic porcelain/Assure Plus group (100%) while the lowest frequency was noted in IPS/Assure Plus group (40%) ( Table 3).

**Table 3:**
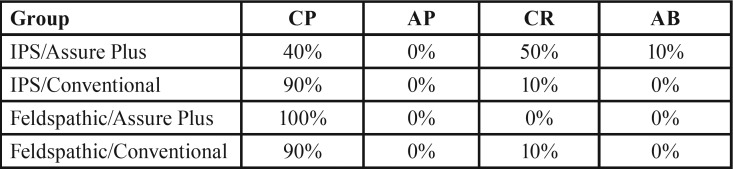
Mode of failure in the groups.

## Discussion

Adequate bond strength in orthodontics is different from that in prosthodontics and operative dentistry. For porcelain repair, higher bond strength values are preferred while in orthodontics, bond strength should not be too high since it might damage the porcelain surface during debonding (2,13).

Karan *et al.* reported that orthodontic appliances exert an approximate 1.5-MPa load on teeth (14). On the other hand, Reynolds in 1975 reported that 5 MPa of bond strength was required to achieve clinical success (15). The clinically acceptable shear bond strength values in other studies have been reported to be 6‒8 MPa (16,17). In another report, ideal bond strength of orthodontic brackets was reported to be 6‒10 MPa (18). However, it should be noted that generalizability of these values to the clinical setting is limited since the bond of orthodontic brackets to ceramics is influenced by several environmental factors (8).

Based on the results of the current study, bond strength in all the four groups was above the minimum required value. Avoka *et al.* prepared feldspathic porcelain surfaces with HF (9.6% for 20 seconds) and silane for bonding and obtained the highest strength (15 MPa), followed by the sandblasting and silane group (13.8 MPa) (19). These values for the same porcelain were reported to be 9.4 MPa in the HF (9.6% for 2 minutes) group and 11.6 MPa in the sandblasting group (5). Sarac *et al.* reported bond strengths of 11.8 MPa for lithium disilicate and 13.8 MPa for feldspathic porcelain when the samples were sandblasted and silanized (10). Smaller values (9.4 MPa) have also been reported after HF etching of feldspathic porcelain for 4 minutes (20). The bond strength value of 5.5‒6.5 MPa was recorded for IPS Classic porcelain after sandblasting and HF etching for 2 minutes with different silanes, with no significant difference between 5% and 9% concentrations of HF (21). These differences are primarily explained by the type of surface preparation method. In addition, different HF concentrations and etching times might influence the bracket bonding results. In the current study, maximum preparation of the surface (including sandblasting and HF etching) was performed. Differences can also be attributed to the use of different tests, thermal cycles, bond strength testing machines, direction of the load applied for bracket debonding, crosshead speed and type of bracket (22). Moreover, use of different ceramics may be responsible for different bond strength values since ceramics are variable in terms of size of particles and their crystalline structure (11). Therefore, bond strength values obtained in this study could not be directly compared with the studies mentioned above.

In the current study, the bond strength of brackets to ceramic surfaces was significantly higher in IPS ceramic/Assure Plus subgroup compared to the two feldspathic porcelain subgroups. In a study by Abu Alhaji *et al.*, the bond strength of brackets to metal-ceramic crowns was higher than that to IPS-Impress porcelain (11). However, in the study by Sarac *et al.*, with all surface conditionings, bond strength value for leucite-reinforced (IPS Empress) was reported to be higher than that for feldspathic porcelain (10), which consistent with the current findings. According to a study by Neis *et al.*, lithium disilicate ceramic has less glass content than feldspathic porcelain; thus, chemical preparation with hydrofluoric acid creates a porous pattern with higher retention due to the dissolution of glass phase (23).

Assessment of ARI scores in the current study showed that except for IPS/Assure Plus subgroup in 90‒100% of samples in the remaining groups no adhesive remained on the porcelain surface; however, this does not mean that the bond strength of adhesive to bracket was higher than that of adhesive to porcelain surface because at the time of debonding, fracture occurred within the porcelain, which indicated that the bond strength of adhesive to porcelain was stronger than the cohesive strength of porcelain and caused fracture within the porcelain structure.

Nonetheless, it should be noted that ARI scores do not exactly represent bond strength. According to O’Brien *et al.*, ARI depends on several factors such as bracket base design and type of adhesive. Thus, mode of failure cannot be predicted based on the bond strength value (24). In debonding of bracket from the porcelain surface, four types of fractures may occur, namely cohesive within the porcelain, adhesive at the porcelain‒adhesive interface, cohesive within the adhesive layer and adhesive at the adhesive‒bracket interface. Mode of failure is important in that it shows possible side effects of debonding on the porcelain surface. Damage to porcelain may necessitate its replacement. Thus, it is extremely important to minimize the risk of damage to porcelain as much as possible.

In the current study, except for IPS/Assure Plus group, which showed 40% cohesive failure within the porcelain, the remaining groups showed 90‒100% cohesive failures, which might be attributed to structural differences of ceramics and higher fracture strength of lithium disilicate ceramic compared to feldspathic porcelain. Although damage to porcelain was seen in a large number of samples in our study, this result cannot be simply generalized to the clinical setting.

In the clinical setting, damage to porcelain surface during bracket debonding rarely occurs (25), probably due to the method of debonding in the clinical setting, which is done by applying peeling load and is different from laboratory tests.

Considering the simple application of Assure Plus universal adhesive compared to the conventional application of silane and primer and the acceptable bond strength obtained between ceramic and bracket, it appears that this adhesive can yield predictable results in orthodontic treatment. Assure Plus All Surface enables a direct bond to different surfaces with adequate strength; however, surface preparation is still required to achieve the highest shear bond strength possible in the samples.

In general, use of Assure Plus universal adhesive in the current study slightly increased the bond strength to lithium disilicate ceramic surfaces compared to the conventional method. However, no significant difference was noted in bond strength between Assure Plus and conventional adhesive to feldspathic ceramic surfaces. In addition, application of Assure Plus was superior to conventional adhesive for bond of brackets to lithium disilicate ceramics since the former caused less damage in the porcelain surface during debonding. Assure Plus is a universal adhesive and can be used for bonding to all types of restorations and enamel even in areas where adequate isolation cannot be achieved. Moreover, it provides adequate bond strength of brackets to porcelain and even decreases the risk of damage to ceramic surfaces during debonding. Thus, it can be a suitable alternative to other bonding resins in orthodontic treatment. Its use is also cost-effective. However, its optimal efficacy for use on other restorative materials must be confirmed in future studies.

In the current study, all the samples were submitted to thermal cycling (2000 cycles) to simulate conditions of oral environment. In restorative dentistry, water storage and thermocycling are used as artificial aging methods. When bonded materials with different coefficients of thermal expansion and thermal conductivities are submitted to temperature variations, thermal stress is produced at the bonded interface. This may result in a decrease in mechanical properties of the bonding resin and a decrease in bond strength. In addition, hydrolytic degradation of the interface components might contribute to the decreased strength (26). In orthodontic studies, 500‒6000 thermal cycles were used (10,19,20,27). However, a recent study revealed that use of at least 7000 cycles is necessary to bring about a significant reduction of bracket bond strength to ceramic (28). More studies are required to evaluate the bonding ability of Assure Plus under a larger number of thermocycling.

## Conclusions

Assure Plus provides a strong bond between ceramic and brackets. It has a simple application process and decreases the risk of damage to lithium disilicate ceramics during debonding compared to conventional adhesive. Thus, its use is recommended in orthodontic treatment of adults with ceramic restorations.
